# Correlation between Eye Movements and Asthenopia: A Prospective Observational Study

**DOI:** 10.3390/jcm11237043

**Published:** 2022-11-28

**Authors:** Ziyuan Liu, Kaiyun Zhang, Shuang Gao, Jiarui Yang, Weiqiang Qiu

**Affiliations:** 1 Department of Ophthalmology, Peking University Third Hospital, 49 North Garden Road, Haidian District, Beijing 100191, China; 2 Beijing Key Laboratory of Restoration of Damaged Ocular Nerve, Peking University Third Hospital, Beijing 100191, China; 3 College of Optometry, Peking University Health Science Center, Beijing 100038, China

**Keywords:** visual fatigue, asthenopia, eye movements, accommodation

## Abstract

Purpose: To analyze the correlation between eye movements and asthenopia so as to explore the possibility of using eye-tracking techniques for objective assessment of asthenopia. Methods: This prospective observational study used the computer visual syndrome questionnaire to assess the severity of asthenopia in 93 enrolled college students (age 20–30) who complained about asthenopia. Binocular accommodation and eye movements during the reading task were also examined. The correlations between questionnaire score and accommodation examination results and eye movement parameters were analyzed. Differences in eye movement parameters between the first and last reading paragraphs were compared. The trends in eye movement changes over time were observed. Results: About 81.7% of the subjects suffered from computer visual syndrome. Computer visual syndrome questionnaire total score was positively correlated with positive relative accommodation (*p* < 0.05). In the first reading paragraph, double vision was positively correlated with unknown saccades (all *p* < 0.05). Difficulty focusing at close range was positively correlated with total fixation duration, total visit duration, and reading speed (all *p* < 0.05). Feeling that sight was worsening was positively correlated with regressive saccades (*p* < 0.05). However, visual impairment symptoms were not significantly correlated with any accommodative function. In a total 20 min reading, significantly reduced eye movement parameters were: total fixation duration, fixation count, total visit duration, visit count, fixation duration mean, and reading speed (all *p* < 0.01). The eye movement parameters that were significantly increased were: visit duration mean and unknown saccades (all *p* < 0.001). Conclusion: Eye tracking could be used as an effective assessment for asthenopia. Among the various eye movement parameters, a decrease in fixation duration and counts may be one of the potential indicators related to asthenopia.

## 1. Background

Asthenopia, also known as visual fatigue, is characterized by symptoms related to close work, such as blurred vision, diplopia, dry eyes, and headache. It can be divided into three categories, i.e., visual disturbance, ocular irritation, and extraocular symptoms. As electronic screen terminals become an indispensable tool in daily life, the incidence of asthenopia has increased. In the past 5 years, the incidence rate of asthenopia in adults has been reported to be 53.3–82.4% [1]. As a result, asthenopia has become a serious public health problem as it affects life quality, reduces work efficiency, endangers mental health, and increases the financial burden associated with medical treatment.

Clinically, subjective symptoms, such as eye pain and blurred vision, are essential for diagnosing asthenopia. However, there is a lack of objective measurements for diagnosis. Accommodation examination has been previously used; however, its detection rate was low [2]. Therefore, other objective biological signals, such as electroencephalogram, electromyography, and electrooculography, have been used to determine the severity of asthenopia [3,4]. Still, subjects must wear the device on their head during the experiment, which might make them uncomfortable, thus affecting the visual tasks. In addition, noise artifact and complex biological signals make the results hard to interpret. Consequently, new reliable and sensitive methods for assessment of asthenopia are urgently needed.

Eye movement tracker is a rapidly developing technology that has the potential to become an objective and repeatable means for evaluating asthenopia. The principle of the eye movement tracker is to accurately determine the subject’s gaze direction by measuring the position of the corneal reflection of infrared light relative to the pupil. It can also be used for accurate and real-time eye tracking in various visual tasks [5]. The modes of eye movement mainly include fixation and saccade. Fixation is the only eye movement that can extract information from the target. A saccade is the rapid movement between two adjacent fixations under the movement of extraocular muscles, i.e., the process of the clearest visual direction of the fovea of the retina moving from one position to another, which usually lasts for 30–50 ms [6]. Previous studies have shown that fixation stability, saccade latency, and saccade amplitude can greatly change in ophthalmic diseases, such as oblique amblyopia, glaucoma, and ametropia [7,8,9]. In addition, when healthy people are driving, their eye movements, such as saccade speed, may significantly change with the emergence of mental or physical fatigue [10,11]. While a limited number of studies on the association of asthenopia and eye movement have been reported in recent years [12,13,14,15], the efficacy of eye movement for objective assessment of asthenopia needs to be further examined.

This study preliminarily explored the correlation between different eye movement indexes and asthenopia symptoms and the possibility of using eye movement tracking in objective and measurable evaluation for asthenopia.

## 2. Methods

### Study Design and Participants

This prospective observational study enrolled 93 healthy college students aged between 20 and 30. Prior to participating in the study, all applicants underwent an ophthalmic screening examination by one clinician, including visual acuity, noncycloplegic manifest refraction, slit lamp examination, fundus examination, and cover test. The inclusion criteria were: (1) complaining about asthenopia; (2) best-corrected vision of 20/20 for both eyes. The exclusion criteria were: (1) eye diseases other than refractive errors; (2) myopia exceeding 9.0 D, hyperopia exceeding 1.0 D, anisometropia exceeding 2.0 D, or astigmatism over 2.0 D; (3) and previous eye surgeries.

The study was conducted according to the Declaration of Helsinki principles, and ethical approval was obtained from the ethics committee of Peking University Third Hospital before initiation of the study (M2018093). Informed consent was obtained from all participants.

## 3. Procedures

### 3.1. Eye Fatigue Assessment

The computer vision syndrome questionnaire (CVS-Q) was used to evaluate the participants’ symptoms based on their daily computer experience [16]. The frequency and intensity of 16 symptoms were assessed across the following three categories: vision disturbance, including blurred vision, double vision, difficulty focusing for near vision, and colored halos; ocular discomforts, including burning, itching, feeling of a foreign body, tearing, excessive blinking, eye redness, eye pain, and dryness; and extraocular symptom of headache. A rating scale of 0–2 points was used to measure the frequency, with never = 0, occasionally (once a week or sporadic episodes) = 1, often (two to three times a week)/always (almost every day) = 2. The severity was graded on a scale of 1–2 points, with moderate = 1 and intense = 2. The total score was calculated with the following expression (recoding the result of Frequency X Intensity as: 0 = 0; 1 or 2 = 1; 4 = 2):

$$Score=\overset{16}{\underset{i-1}{\sum }}{\left(frequency\;of\;symptomoccurrence\right)}_{i}\times {\left(intensity\;of\;symptom\right)}_{i}.$$

If the CVS-Q score was ≥6, the subject was considered to suffer from computer vision syndrome [16].

### 3.2. Accommodation

Five parameters of accommodating ability were analyzed, including accommodation amplitude (AMP), accommodation accuracy (AR), negative relative accommodation (NRA), positive relative accommodation (PRA), and binocular accommodative facility (BAF). Since in daily life accommodation is generally stimulated under binocular conditions, the measurements are binocular in this study. (1) AMP was examined with a near vision chart that was moved from 40 cm towards the subjects until they reported sustained blurriness, and the inverse of the distance (cm) was recorded as the AMP diopter. (2) AR was tested binocularly through ±0.5 D cross-cylinder to a 40 cm target with horizontal and vertical meridians. Further, +0.25 D lenses were added until the vertical lines appeared clearer, after which the additions were reduced with −0.25 D intervals until the horizontal and vertical lines were equally clear. The least amount of plus was recorded as the AR. (3) Using a 20/30 chart setting 40 cm in front of the subject, NRA was assessed by adding +0.25 D lenses until they reported sustained blurriness, and the amount of plus added was the NRA. (4) Next, −0.25 D lenses were added until they reported sustained blurriness, and the amount of minus added was the PRA. (5) BAF was evaluated by reading the 20/30 chart at 40 cm through a flipper (±2.00 D) in one minute, and the cycles per minute (cpm) were recorded as the BAF. Eye alignment was evaluated via the alternative cover test at 40 cm and was recorded as ortho (no movement of eyes) or tropia/phoria.

### 3.3. Eye Movement Tracking

**Figure 1:**
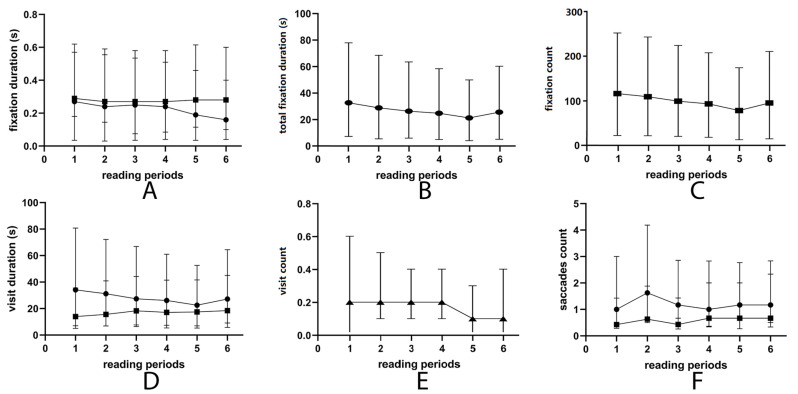
Changes in eye movement parameters during 6 reading periods. (**A**) First fixation duration decreased when reading time increased (dot), and first fixation duration was shorter than fixation duration mean (square) during all reading processes. (**B**,**C**) Total fixation duration (**B**) and fixation count (**C**) decreased during reading. (**D**) Total visit duration (dot) and visit count (triangle) decreased with time, and visit duration mean (square) increased with time. (**E**) Reading speed decreased with time, and no linear correlation was detected. (**F**) Regressive saccade (dot) increased during the second reading process and declined later. Unknown saccade (square) increased with time, but no linear correlation was found.

Infrared eye-tracking equipment (Tobii Pro X3-120 Eye Tracker; Tobii AB Inc., Stockholm, Sweden) was used, and the reading texts were displayed as an 18-point font on a computer (Dell ST2220Mb; Dell Corporation, Round Rock, TX, USA), with a resolution of 1920 × 1200. The participants were seated 65 cm from the screen in an appropriate luminous environment and instructed to keep their body and head steady while reading with their habitual spectacle correction. Before the test, the procedure and details were explained to the participants. The reading texts included six passages containing 185–273 words (an example text is shown in Figure 1). After finishing each passage, the participants were told to press the space key to end that particular passage and answer questions related to comprehension of the texts and subsequently press the space key again to begin the next passage. In the beginning, during intervals of two passages and ending, a red point appeared in the middle of the screen to make subjects keep gazing. The total duration of the reading track test was approximately 20 min.

### 3.4. Eye Movement Analysis

To quantify the subjects’ eye movement, the area of interest (AOI) was defined as a rectangle-area-covered reading text. These following parameters were automatically taken from Tobii Pro Studio software (version 3.4.8, Tobii Technology AB, Stockholm, Sweden), including time to first fixation (duration before fixating on the AOI), fixation before (time of fixation before fixating on the AOI), first fixation duration (duration of the first fixation on the AOI), fixation duration mean (average duration of all fixations on the AOI), total fixation duration (sum of all the fixation durations on the AOI), fixation count (time of fixation on the AOI), visit duration mean (average of all visits on the AOI, where a visit referred to a period from the first fixation on the AOI until the first fixation moving away from the AOI), total visit duration (sum of all visit durations), and visit count (times of visits). The reading speed (characters per second), regressive saccade (numbers of saccades backward per line), and unknown saccade (numbers of meaningless saccades per line) were also analyzed.

### 3.5. Cover Test

The alternate cover test of each eye was practiced with subjects wearing habitual glasses and fixating 20/30 chart at 40 cm.

## 4. Statistical Analysis

Descriptive statistics were summarized as the mean ± standard deviation (SD). Shapiro–Wilk test was used for all ocular and non-ocular parameters to determine whether the data were normally distributed. The nonparametric correlations were analyzed using Spearman’s correlation. Linear regression was conducted for continuous variables. Statistical analyses were performed using SPSS software (version 23; IBM Corp., Armonk, NY, USA), and *p*-value < 0.05 was considered as statistical significance.

## 5. Results

### 5.1. Demographic Characteristics

Among the ninety-five participants who were screened, two failed to complete the tests. Finally, 31 male and 62 female participants (mean age 24.09 ± 2.57 years old) were included in the study.

All 93 participants spent at least 4 h a day working on a computer, and 84 (of 93) participants spent over 5 h daily. Seventy-six subjects had CVS-Q scores of ≥6 and were thus considered to suffer from computer vision syndrome. The frequency of each symptom is listed in Table S1. All the participants either had myopia or emmetropia with spherical equivalent (SE) ranging from −8.5 D to 0.75 D (binocular mean SE −3.72 ± 1.72 D). Only four (4.3%) participants had an abnormal accommodating function. Details of accommodative amplitude, accommodative accuracy, positive and negative relative accommodation, and accommodative facility are listed in Table S2. The eye movement features regarding fixation, visit duration, saccade, and reading speed in the first paragraph reading were recorded in Table S3. The metrics of other paragraphs were used to analyze the changes in eye movement during reading.

### 5.2. Correlations of Asthenopia and Accommodation/Eye Movement

There were no correlations between the total CVS-Q score and any accommodation or eye movement measurement despite a weak correlation with positive relative accommodation (r = 0.206, *p* = 0.047) (Table 1).

Blurred vision, double vision, difficulty focusing at close range, increased sensitivity to light, colored halos around objects, and feeling of sight worsening were categorized as a visual disturbance. Blurred vision was not correlated with any accommodation and eye movement metrics. Double vision was not correlated with any accommodation measurements and most eye movement metrics, while a weak correlation was found with unknown saccade (r = 0.217, *p* = 0.041). Difficulty focusing at close range was correlated with fixation duration (r = 0.235, *p* = 0.023), visit duration (r = 0.253, *p* = 0.014), and reading speed (r = 0.237, *p* = 0.022). This symptom had no relationship with accommodation. Feeling of sight worsening was correlated with regressive saccade (r = 0.270, *p* = 0.011; Table 2). 

Symptoms of ocular surface irritations, such as foreign body sensation, tearing, excessive blinking, eye pain, heavy eyelids, and dryness, were not correlated with accommodation and eye movement measurements. Burning was correlated with fixation count (r = 0.278, *p* = 0.007), regressive saccade (r = 0.243, *p* = 0.022), and unknown saccade (r = 0.249, *p* = 0.019). Eye redness was correlated with mean visit duration (r = 0.25, *p* = 0.016) and time to first fixation (r = −0.215, *p* = 0.038). Both itching and tearing were correlated with positive relative accommodation (r = −0.265, *p* = 0.011 for itching and r = 0.261, *p* = 0.011 for tearing; Table 3). Headache was not related to any accommodation or eye movement measurements. 

### 5.3. Eye Movement Alterations during Reading

Eye movement metrics in six reading paragraphs are recorded in Table 4. The parameters of fixation are shown in Figure 1A,B. The mean fixation duration was always longer than the time to first fixation (Z = −11.25, *p* < 0.001). Time to first fixation, fixation duration, and fixation count decreased with reading time.

The changes in total visit duration, mean visit duration, and visit count are shown in Figure 1C. They all had significant linear correlations with time (Table 5). The mean visit duration slightly increased with the time. The reading speed, regressive saccade, and unknown saccade did not change with time (Figure 1D,E). None of them were significantly correlated with reading time (Table 5).

We compared eye movement measurements from the beginning (the first paragraph) and the end (the last paragraph) of the 20 min reading (Table 6). Most metrics showed significant alterations, including mean fixation duration (Z = −2.711, *p* = 0.007), total fixation duration (Z = −5.0, *p* < 0.001), fixation count (Z = −4.075, *p* < 0.001), mean visit duration (Z = −2.728, *p* = 0.006), total visit duration (Z = −4.945, *p* < 0.001), visit count (Z = −3.998, *p* < 0.001), reading speed (Z = −3.357, *p* = 0.001), and unknown saccade (Z = −3.914, *p* < 0.001). Mean visit duration, regressive saccade, and unknown saccade increased during the reading (Table 4).

## 6. Discussion and Conclusions

A survey of 1022 Chinese college students revealed that using a computer for more than 3 h per day was one of the risk factors for asthenopia [17]. Another study from the United States reported similar results, arguing that asthenopia occurred in 90% of users exposed to computers for more than three hours a day [18]. In the present study, all the participants used computers for at least 4 h per day, and 81.7% had asthenopia. Dry eye was the most common symptom (90.3%), followed by decreased vision (75.3%), itchy eyes (73.1%), and blurred vision (61.3%). In 2021, Zayed et al. used the same questionnaire (CVS-Q) to investigate 108 IT practitioners and found an asthenopia rate of 82.41% [19]. In addition, Touma et al. found that 67.8% of 457 American college students reported at least one symptom of asthenopia, among which blurred vision was the most common, accounting for 27.0% [20].

Compared with asthenopia, which occurred in 81.7% of the cases, only four (4.3%) participants had accommodation dysfunction in our study. It seemed that accommodation dysfunction could not reflect the status of asthenopia sensitively. In the present study, the CVS-Q total score was only correlated with positive relative accommodation (PRA). Positive relative accommodation represents the ability of the accommodative system to see near target under maximum strain, as measured by the addition of negative lenses in front of both eyes on a far correction basis [21]. The affected positive relative regulation indicated that the ability to regulate tension worsened. Wang et al. used a different questionnaire; nevertheless, they obtained a result consistent with ours, suggesting that asthenopia was significantly positively correlated with positive relative accommodation. They also found that asthenopia was significantly negatively correlated with negative relative accommodation, while there was no significant correlation with accommodation sensitivity [12].

The association of visual disturbance and accommodation was tested. Previous studies suggested that asthenopia was largely due to uncorrected or inadequate accommodation of ametropia [22] and that college students with at least one ocular or visual complaint had significantly less accommodative amplitude and were nearer the point of convergence than those without fatigue [23]. Still, our results revealed no significant correlation between accommodation function and the scores of visual symptoms, such as difficulty in seeing things at close range. It seems that, in healthy young people, the index of accommodative function cannot always sensitively reflect the symptoms of visual impairment. Accommodation was closely related to symptoms such as near vision difficulties only in the population meeting the diagnostic criteria of accommodative dysfunction [24]. Moreover, the accuracy of optometry or accommodation examination is 0.25 D. Compared with the measurement accuracy of 0.4° of the eye tracker and the sampling frequency of 120 Hz, the accuracy of the results of accommodation examination may be limited.

Moreover, we found significant positive correlations between the symptoms of difficulty in seeing near things and the total duration of fixation, total interview time, and reading speed, although these correlations were not very strong. These results suggest that, the more severe the symptoms of difficulty in seeing things at a close distance, the longer it takes to locate the target location and complete reading. We found a positive correlation between perceived decreased visual acuity and the number of regressive saccades, defined as the number of saccades in each line of text that was read in the opposite direction (from right to left). A regressive saccade is a process of returning to the previous text to retrieve information, which breaks the original semantics and syntax. In reading scenes, about 10–25% of eye movements belong to regressive saccade [25]. Regressive saccades are very important for reading and for the process of retrieving information [26]. The positive correlation between visual disturbance score and regressive saccade indicated that the increase in regressive saccade would compensate for the missing text information during reading when the visual signal is insufficient. There was a weak positive correlation between double vision and the number of unknown saccades, which referred to the number of saccades unrelated to acquisition of text information in each line of text. Previous studies reported double vision to be associated with eye diseases, such as strabismus and glaucoma [27,28]. In this study, the positive correlation between unknown saccades and double vision implied the probability of gaze position deviation caused by increased object image ghosting.

Second, the association between ocular disturbance and accommodation was analyzed, revealing no significant correlation. The eye discomfort in asthenopia is mainly caused by dry eye syndrome [18,29], which is characterized by tear film instability, tear hyperosmolality, ocular surface inflammation, and corneal nerve abnormalities. In addition, uneven tear film distribution may also lead to visual fluctuation or blurring [30], which affects performance in visual tasks. Van and colleagues found that, among older adults, those with dry eye were more likely to experience reading difficulties; however, there was no significant decrease in reading speed compared with normal controls [31]. Nonetheless, real-time tear film cannot be detected during reading. In the present study, burning, itching, tearing, and red-eye symptoms were found to be significantly correlated with the number of fixation points and the number of regressive and unknown saccades.

During the reading process that lasted some 20 min, the eye movement parameters in the first reading material and the last reading material were compared, revealing that a number of eye movement parameters significantly decreased, including the average duration of fixation, the total duration of fixation, the number of fixation counts, the total visit time, the number of visits, and the reading speed. Moreover, a number of eye movement parameters significantly increased, including average visit time and the number of unknown saccades.

“Visit” includes the process from the first fixation appearing in the region of interest to the first fixation moving out of the region of interest, i.e., the sum of all fixations and saccades in this range. Katz et al. found that an increase in the number and duration of visits to specific regions of interest was associated with increased visual attention [32]. In the current study, both the number of visits and the total duration of visits decreased, but the average duration of visits increased, which may be related to the increase in attention concentration.

As mentioned above, fixation duration and frequency are important predictors of reading fluency [33], and prolonged fixation duration indicates a decline in text readability [34]. Our results revealed that, as reading progressed, sum and mean fixation duration decreased, and the number of fixation count in the area of interest also decreased, implying lower and shorter fixation. Similarly, in a non-reading scenario, Wang et al. used an eye tracker to record 25 subjects watching videos on a computer and found decreased eye movements (fixation number and fixation duration during the first hour) and increased unknown saccades [14]. Nevertheless, in another study, the eye movements of 38 subjects during a 40 min simulated computer task were observed and the changes in mean fixation time were not significant, while the number of unknown saccades increased [35]. Smith et al. observed eye movements in 14 glaucoma patients during reading in 2014 and found more meaningless saccades in poor vision eyes than in good vision eyes. Moreover, the increase in meaningless saccades slowed down the reading speed [26].

This study has a few limitations. First, we only included young people. In further research, different age groups should be recruited, as well as an incipient presbyopia population or people from different educational backgrounds or pseudophakic eyes. Moreover, the sample size should be expanded since eye-tracking measurements of a reading task could vary a great deal between different individuals, which is often influenced by multiple factors. Last, dry eye should be examined for participants as ocular surface signs could provide more information.

To sum up, in a short reading time of about 20 min, several eye-tracking parameters altered. The visit time and frequency changed, the number and time of fixation decreased, the unknown saccade increased, and the reading speed slowed down. Compared with studies on longer visual tasks reported over recent years [12,15], this study further found that the eye movement changes can happen over a very short period of reading (20 min). As in such a relatively short time, asthenopia may not arise so significantly as to be detected by the subject; this demonstrates the potential of eye-tracking technology for sensitive assessment of asthenopia. Among the above eye movement parameters, the decrease in fixation duration and counts may be one of the potential indicators related to asthenopia.

## Figures and Tables

**Table 1:** Correlations between total CVS-Q score and accommodation and eye movement.

	CVS-Q Score	
	*r*	*p*
Accommodation amplitude (D)	0.026	0.807
Accommodative response (D)	0.076	0.471
Negative relative accommodation (D)	−0.013	0.901
Positive relative accommodation (D)	0.206	0.047 *
Accommodative facility (cmp)	−0.148	0.155
Strabismus	0	0.998
Time to first fixation (s)	−0.03	0.776
Fixations Before	−0.059	0.576
First Fixation Duration (s)	−0.034	0.743
Fixation Duration Mean (s)	−0.05	0.634
Total Fixation Duration (s)	0.154	0.14
Fixation count	0.201	0.053
Visit Duration Mean (s)	0.032	0.759
Total visit duration (s)	0.174	0.095
Total visit count	0.014	0.896
Reading speed (s^−1^)	0.153	0.142
Regressive saccade (per line)	0.194	0.068
Unknown saccade (per line)	0.148	0.168

*: *p* < 0.05; *r*: Spearman coefficient; CVS-Q: computer vision syndrome questionnaire.

**Table 2:** Correlations of visual disturbance and accommodation and eye movement.

	Blurred Vision		Double Vision		Difficulty Focusing for Near Vision		Increased Sensitivity to Light		Colored Halos		Feeling of Sight Worsening	
	*r*	*p*	*r*	*p*	*r*	*p*	*r*	*p*	*r*	*p*	*r*	*p*
Accommodation amplitude (D)	0.032	0.757	−0.009	0.929	−0.154	0.142	0.043	0.68	0.07	0.502	0.008	0.94
Accommodative response (D)	−0.013	0.9	0.037	0.723	0.014	0.897	0.111	0.291	0.016	0.881	−0.02	0.848
Negative relative accommodation (D)	0.023	0.828	−0.065	0.533	−0.106	0.312	0.01	0.926	−0.025	0.812	−0.019	0.855
Positive relative accommodation (D)	0.095	0.363	−0.022	0.832	0.109	0.298	0.032	0.762	0.092	0.38	0.138	0.188
Accommodative facility (cmp)	−0.036	0.733	−0.098	0.349	0.033	0.751	0.034	0.743	−0.128	0.222	−0.05	0.631
Strabismus	−0.049	0.655	0.083	0.454	0.063	0.570	0.099	0.372	0.041	0.709	−0.151	0.171
Time to first fixation (s)	0.027	0.794	0.131	0.21	0.279	0.007 **	−0.142	0.175	−0.129	0.22	0.083	0.431
Fixations Before	0.083	0.428	0.163	0.118	0.093	0.375	−0.165	0.115	−0.09	0.392	0.058	0.582
First Fixation Duration (s)	−0.047	0.655	−0.061	0.563	−0.115	0.273	−0.138	0.186	−0.018	0.867	−0.085	0.419
Fixation Duration Mean(s)	−0.104	0.322	−0.113	0.279	0.107	0.308	0.098	0.352	−0.231	0.026 *	0.032	0.763
Total Fixation Duration(s)	0.084	0.425	−0.014	0.892	0.235	0.023 *	0.142	0.173	−0.027	0.796	0.114	0.274
Fixation count	0.186	0.074	0.067	0.522	0.202	0.052	0.135	0.199	0.066	0.527	0.143	0.171
Visit Duration Mean(s)	0.031	0.765	−0.107	0.305	0.157	0.133	0.11	0.292	−0.066	0.533	−0.092	0.381
Total visit duration(s)	0.127	0.227	−0.019	0.856	0.253	0.014 *	0.112	0.284	−0.051	0.63	0.128	0.223
Total visit count	0.038	0.719	0.116	0.27	−0.022	0.834	−0.056	0.592	−0.024	0.822	0.146	0.162
Reading speed (s^−1^)	0.145	0.166	0.029	0.783	0.237	0.022 *	0.083	0.43	−0.079	0.454	0.159	0.129
Regressive saccade (per line)	0.166	0.12	−0.02	0.852	0.029	0.788	0.107	0.319	0.173	0.105	0.27	0.011 *
Unknown saccade (per line)	0.169	0.113	0.217	0.041 *	0.067	0.533	−0.027	0.801	−0.066	0.536	0.066	0.536

*: *p* < 0.05; **: *p* < 0.01; *r*: Spearman coefficient.

**Table 3:** Correlations of ocular irritation and accommodation and eye movement.

	Burning		Itching		Feeling of a Foreign Body		Tearing		Excessive Blinking		Eye Redness		Eye Pain		Heavy Eyelids		Dryness	
	*r*	*p*	*r*	*p*	*r*	*p*	*r*	*p*	*r*	*p*	*r*	*p*	*r*	*p*	*r*	*p*	*r*	*p*
AA (D)	−0.035	0.737	−0.027	0.798	−0.125	0.234	−0.028	0.789	0.054	0.606	−0.06	0.568	−0.033	0.755	0	0.997	0.052	0.623
AR (D)	0.168	0.108	0.104	0.324	0.019	0.86	0.066	0.531	−0.07	0.506	−0.038	0.716	0.11	0.296	0.075	0.474	0.01	0.928
NRA (D)	−0.011	0.92	−0.041	0.701	−0.073	0.485	−0.07	0.506	−0.013	0.898	−0.051	0.63	−0.104	0.322	−0.046	0.662	−0.12	0.254
PRA (D)	0.136	0.193	−0.265	0.011 *	0.18	0.085	0.261	0.011 *	0.163	0.119	0.171	0.101	0.183	0.08	0.032	0.763	0.086	0.41
AF (cmp)	−0.008	0.942	0.045	0.671	−0.149	0.155	−0.042	0.693	−0.2	0.054	−0.092	0.383	0.026	0.807	0.071	0.5	−0.1	0.34
Strabismus	−0.136	0.219	−0.145	0.189	−0.094	0.396	0.111	0.317	0.130	0.239	0.050	0.649	−0.101	0.360	−0.094	0.393	−0.062	0.575
FF (s)	0.016	0.879	−0.115	0.273	−0.146	0.164	−0.16	0.111	0.186	0.074	−0.215	0.038 *	0.009	0.931	−0.005	0.961	0.062	0.556
FB	0.013	0.903	−0.241	0.021 *	−0.173	0.097	−0.18	0.074	0.13	0.213	−0.151	0.149	0.008	0.94	−0.085	0.42	−0.028	0.789
FFD (s)	0.058	0.579	0.087	0.409	0.094	0.368	0.042	0.69	−0.043	0.68	0.102	0.332	0.101	0.334	−0.035	0.742	−0.056	0.592
FDM (s)	−0.054	0.61	0.141	0.179	−0.041	0.693	0.02	0.846	−0.038	0.716	0.042	0.687	0.105	0.319	0.011	0.917	−0.172	0.1
TFD (s)	0.131	0.209	0.147	0.161	0.051	0.626	0.005	0.965	0.004	0.967	0.003	0.975	0.092	0.38	0.094	0.372	−0.079	0.45
FC	0.278	0.007 **	0.031	0.766	0.008	0.941	0.004	0.967	0.015	0.886	−0.059	0.577	0.073	0.485	0.124	0.236	0.004	0.97
VDM (s)	−0.081	0.442	−0.117	0.265	−0.068	0.52	0.19	0.067	−0.026	0.808	0.25	0.016 *	0.117	0.263	0.03	0.774	−0.094	0.368
TVD (s)	0.162	0.12	0.115	0.274	0.032	0.763	0.005	0.964	0.024	0.817	0.026	0.806	0.102	0.33	0.125	0.231	−0.05	0.636
TVC	0.14	0.181	0.132	0.21	0.031	0.766	−0.20	0.047 *	−0.013	0.898	−0.271	0.009 **	−0.064	0.54	0.058	0.583	0.071	0.502
RS (s^−1^)	0.135	0.196	0.117	0.265	0.011	0.92	0.014	0.893	−0.023	0.823	0.015	0.888	0.088	0.399	0.074	0.481	−0.057	0.586
RS (per line)	0.243	0.022 *	0.029	0.788	0.029	0.786	0.057	0.593	0.101	0.345	0.078	0.468	0.119	0.267	−0.068	0.524	0.075	0.484
US (per line)	0.249	0.019 *	0.016	0.884	−0.043	0.69	−0.084	0.435	0.047	0.663	−0.097	0.365	0.171	0.108	0.084	0.435	0.003	0.974

AA: accommodation amplitude, AR: accommodative response, NRA: negative relative accommodation, PRA: positive relative accommodation, AF: accommodative facility, FF: time to first fixation, FB: fixations before, FFD: first fixation duration, FDM: fixation duration mean, TFD: total fixation duration, FC: fixation count, VDM: visit duration mean, TVD: total visit duration, TVC: total visit count, RS: reading speed, RS: regressive saccade, US: unknown saccade. * *p* < 0.05, ** *p* < 0.01; *r* Spearman coefficient.

**Table 4:** Eye movement parameters in reading processes.

	Reading 1	Reading 2	Reading 3	Reading 4	Reading 5	Reading 6
Time to first fixation (s)	0(0)	0(0)	0(0)	0(0)	0(0)	0(0)
Fixations Before	0(0)	0(0)	0(0)	0(0)	0(0)	0(0)
First Fixation Duration (s)	0.27(0.21)	0.24(0.22)	0.25(0.11)	0.24(0.12)	0.19(0.20)	0.16(0.18)
Fixation Duration Mean (s)	0.29(0.08)	0.27(0.08)	0.27(0.08)	0.27(0.08)	0.28(0.09)	0.28(0.08)
Total Fixation Duration (s)	32.72(19.77)	28.9(16.15)	26.36(16.81)	24.81(13.83)	21.28(11.52)	25.63(14.14)
Fixation count	116(42)	109(46.5)	99(46)	93(39.5)	78(30.5)	95(35)
Visit Duration Mean (s)	13.97(12.68)	15.62(16.47)	18.3(15.31)	17.11(14.64)	17.46(13.64)	18.47(17.27)
Total visit duration (s)	34.12(19.55)	31.24(16.64)	27.37(18.52)	26.09(14.27)	22.51(12.94)	27.22(15.84)
Total visit count	2(2)	2(2)	2(1)	2(1)	1(1)	1(2)
Reading speed (s^−1^)	0.13(0.08)	0.13(0.07)	0.11(0.07)	0.13(0.06)	0.12(0.07)	0.11(0.06)
Regressive saccade (per line)	1.00(1.43)	1.63(1.50)	1.17(1.19)	1(1.20)	1.17(1.05)	1.17(1.00)
Unknown saccade (per line)	0.43(0.86)	0.63(1.12)	0.43(0.83)	0.67(1.01)	0.67(0.93)	0.67(1.33)

Data were recorded as median (IQR) or median (Q1, Q3).

**Table 5:** Regression analysis of eye movement parameters and time.

	B	Constant	*p*
Time to first fixation	−0.020	0.296	0.01
Fixation duration mean	−0.0006	0.279	0.804
Total fixation duration	−1.710	32.60	0.043
total fixation count	−5.829	118.7	0.045
Total visit duration	−1.771	34.29	0.048
Visit duration mean	0.767	14.14	0.041
Visit count	−0.229	2.467	0.042
Reading speed	−0.002	0.129	0.413
Regressive saccade	−0.020	1.258	0.076
Unknown saccade	0.044	0.428	0.127

**Table 6:** Comparison of eye movement between the first and last reading process.

	*Z*	*p*
Time to first fixation (s)	−1.084	0.278
Fixations Before	−0.707	0.48
First Fixation Duration (s)	−1.58	0.114
Fixation Duration Mean (s)	−2.711	0.007 **
Total Fixation Duration (s)	−5	<0.001 ***
Fixation count	−4.075	<0.001 ***
Visit Duration Mean (s)	−2.728	0.006 **
Total visit duration (s)	−4.945	<0.001 ***
Total visit count	−3.998	<0.001 ***
Reading speed (s^−1^)	−3.357	0.001 **
Regressive saccade (per line)	−0.28	0.78
Unknown saccade (per line)	−3.914	<0.001 ***

Wilcoxon test; **: *p* < 0.01;***: *p* < 0.001.

## Data Availability

The data presented in this study are available on request from the corresponding author. The data are not publicly available due to the reason that another research is being conducted based on the data.

## Supplementary Materials

The following supporting information can be downloaded at: https://www.mdpi.com/article/10.3390/jcm11237043/s1, Table S1. Computer vision syndrome questionnaire scores frequency. Table S2. Accommodation examination of all participants. Table S3. Eye movement parameters in first reading process.
