# Supplementary material for: Correlation between Eye Movements and Asthenopia: A Prospective Observational Study
Source: J Clin Med. 2022 Nov 28;11(23):7043. doi: 10.3390/jcm11237043 (PMC9739550; doi:10.3390/jcm11237043)
Supplement: Supplementary file 1 [file jcm-11-07043-s001.zip › jcm-2047614-supplementary.pdf]

Supplementary Table S1. Computer vision syndrome questionnaire scores frequency

| | Scores | | |
|---|---|---|---|
| | 0 | 1 | 2 |
| Burning | 50 ( 53.8% ) | 43 ( 46.2% ) | 0（0%） |
| Itching | 25 ( 26.9% ) | 66 ( 71% ) | 1 ( 1.1% ) |
| Feeling of a foreign body | 39 ( 41.9% ) | 53 ( 57% ) | 1 ( 1.1% ) |
| Tearing | 35 ( 37.6% ) | 55 ( 59.1% ) | 3 ( 3.2% ) |
| Excessive blinking | 50 ( 53.8% ) | 36 ( 38.7% ) | 7 ( 7.5% ) |
| Eye redness | 45 ( 48.4% ) | 45 ( 48.4% ) | 3 ( 3.2% ) |
| Eye pain | 49 ( 52.7% ) | 43 ( 46.2% ) | 1 ( 1.1% ) |
| Heavy eyelids | 70 ( 75.3% ) | 21 ( 22.6% ) | 2 ( 2.2% ) |
| Dryness | 9 ( 9.7% ) | 67 ( 72% ) | 17 (18.3% ) |
| Blurred vision | 36 ( 38.7% ) | 48 ( 51.6% ) | 9 ( 9.7% ) |
| Double vision | 54 ( 58.1% ) | 36 ( 38.7% ) | 3 ( 3.2% ) |
| Difficulty focusing for near vision | 72 ( 77.4% ) | 18 ( 19.4% ) | 3 ( 3.2% ) |
| Increased sensitivity to light | 40 ( 43% ) | 47 ( 50.5% ) | 6 ( 6.5% ) |
| Colored halos | 68 ( 73.1% ) | 25 ( 26.9% ) | 0（0%） |
| Feeling of sight worsening | 23 ( 24.7% ) | 64 ( 68.8% ) | 6 ( 6.5% ) |
| Headache | 52 ( 55.9% ) | 39 ( 41.9% ) | 2 ( 2.2% ) |

Supplementary Table S2. Accommodation examination of all participants

| | Median (InterQuartile Range, IQR) | (Q1, Q3) |
|---|---|---|
| Accommodation amplitude (D) | 10.0(2.0) | (8.0, 10.0) |
| Accommodative response (D) | 0 (0.5) | (-0.25, 0.25) |
| Negative relative accommodation (D) | 1.75(1.25) | (1.00, 2.25) |
| Positive relative accommodation (D) | -2.00(1.38) | (-2.50, -1.13) |
| Accommodative facility (cmp) | 10(5) | (8, 13) |

Supplementary Table S3. Eye movement parameters in first reading process

| | Median (InterQuartile Range, IQR) | Q1, Q3 |
|---|---|---|
| Time to first fixation (s) | 0(0) | (0,0) |
| Fixations Before | 0(0) | (0,0) |
| First Fixation Duration (s) | 0.27(0.21) | (0.10, 0.30) |
| Fixation Duration Mean(s) | 0.29(0.08) | (0.25, 0.34) |
| Total Fixation Duration(s) | 32.72(19.77) | (24.89, 45.93) |
| Fixation count | 116(42) | (94.00, 139.50) |
| Visit Duration Mean(s) | 13.97(12.68) | (8.88, 21.86) |
| Total visit duration(s) | 34.12(19.55) | (27.12, 47.32) |
| Total visit count | 2(2) | (2,4) |
| Reading speed ($s^{-1}$) | 0.13(0.075) | (0.10, 0.18) |
| Regressive saccade（per line） | 1(1.43) | (0.57, 2.00) |
| Unknown saccade (per line) | 0.43(0.86) | (0.14, 1.00) |
